# Key Intervention Characteristics in e-Health: Steps Towards Standardized Communication

**DOI:** 10.1007/s12529-016-9630-3

**Published:** 2017-04-12

**Authors:** Bridgette M. Bewick, Steven J. Ondersma, Mette T. Høybye, Oskar Blakstad, Matthijs Blankers, Håvar Brendryen, Pål F. Helland, Ayna B. Johansen, Paul Wallace, Kristina Sinadinovic, Christopher Sundström, Anne H Berman

**Affiliations:** 1 University of Leeds, Leeds, UK; 2 Merrill-Palmer Skillman Institute and Department of Psychiatry & Behavioral Neurosciences, Wayne State University, Detroit, MI UK; 3 Interdisciplinary Research Unit, Elective Surgery Center, Regional Hospital Silkeborg, Silkeborg, Denmark; 4 Department of Clinical Medicine, Aarhus University, Aarhus, Denmark; 5 Explorable AS, Kristiansand, Norway; 6 Trimbos Institute The Netherlands Institute of Mental Health and Addiction, Utrecht, The Netherlands; 7 Arkin Mental Health Care, Amsterdam, The Netherlands; 8 Department of Psychiatry, Academic Medical Centre, University of Amsterdam, Amsterdam, The Netherlands; 9 The Norwegian Centre for Addiction Research, Institute of Clinical Medicine, Faculty of Medicine, University of Oslo, Oslo, Norway; 10 Blue Cross Addiction Clinic, Oslo, Norway; 11 Research Department of Primary Care and Population Health, University College London, London, UK; 12 Department of Clinical Neuroscience, Center for Psychiatry Research, Karolinska Institutet, Stockholm, Sweden; 13 Stockholm Center for Dependency Disorders, Stockholm, Sweden

**Keywords:** e-Health intervention, Technology, Behavior, Guideline, Standardized reporting

## Abstract

**Purpose:**

This paper reports expert opinion on e-health intervention characteristics that enable effective communication of characteristics across the diverse field of e-health interventions. The paper presents a visualization tool to support communication of the defining characteristics.

**Methods:**

An initial list of e-health intervention characteristics was developed through an iterative process of item generation and discussion among the 12 authors. The list was distributed to 123 experts in the field, who were emailed an invitation to assess and rank the items. Participants were asked to evaluate these characteristics in three separate ways.

**Results:**

A total of 50 responses were received for a response rate of 40.7%. Six respondents who reported having little or no expertise in e-health research were removed from the dataset.

Our results suggest that 10 specific intervention characteristics were consistently supported as of central importance by the panel of 44 e-intervention experts. The weight and perceived relevance of individual items differed between experts; oftentimes, this difference is a result of the individual theoretical perspective and/or behavioral target of interest.

**Conclusions:**

The first iteration of the visualization of salient characteristics represents an ambitious effort to develop a tool that will support communication of the defining characteristics for e-health interventions aimed to assist e-health developers and researchers to communicate the key characteristics of their interventions in a standardized manner that facilitates dialog.

## Introduction

The use of e-health technologies to deliver interventions continues to evolve, with interventions becoming increasingly diverse in content, target, intensity, and form of technology. There is a clear advantage in the establishment of standards for characterizing e-health interventions, particularly for later systematic reviews and meta-analyses that may seek to identify characteristics most closely associated with outcomes. Existing reviews, e.g., [1–4] select interventions that share commonalities and typically do not seek to establish dialog across the diversity of e-health interventions. The language used to describe even seemingly similar interventions varies, making it difficult to uncover the commonalities and/or differences across the field of e-health interventions.

Efforts have been made to standardize reporting of e-health interventions for disciplines [5] and journals (e.g., [6, 7]). This standardization of dissemination of findings has facilitated consideration of mechanisms of change. The developing literature for evaluating and reporting studies on e-health interventions is working towards a consensus of language and content for reporting effectiveness studies in e-health (e.g., [8, 9]). Recommended reporting criteria include methodological factors such as usability and content testing, extent of user feedback, cost assessment, and limitations for delivery at scale, along with a number of recommended criteria describing the intervention itself (e.g., [7]). Further, within disciplines, efforts are ongoing to create complete micro-level taxonomies (e.g., [2, 5, 10, 11]). However, there is a critical need to also capture key intervention characteristics to facilitate communication among intervention developers; the existing, relatively comprehensive taxonomies are far too long to allow rapid communication of key elements. However, to date, there has been no attempt to systematically identify a limited list of the intervention characteristics that developers and researchers see as the most salient from among the tremendous number of possible descriptors. Doing so could allow for standardized characterization of e-health interventions in a way that is sufficiently detailed to be useful yet streamlined enough to be adopted for regular use outside of in-depth reviews. The establishment of a standard, highly salient limited descriptor set could also facilitate consistent reporting of key intervention characteristics.

This paper aims to: (1) report expert opinion regarding a comprehensive list of e-health intervention characteristics, with an emphasis on identification of a limited set that could enable effective and comprehensible communication of characteristics across the diverse field of e-health interventions and (2) present a visualization tool to support communication of the defining characteristics.

## Methods and Materials

The list of intervention characteristics was developed through an iterative process of item generation and discussion among the 12 authors. A longer initial list—attempting to represent all possible characteristics on which e-health interventions could differ—was reduced by elimination of overlapping characteristics, and unclear characteristics were clarified or removed. The 12 authors reviewed and revised the list using an online survey. Upon reaching consensus, the final version of the survey consisted of 25 specific and distinct intervention characteristics (see Table 1).

**Table 1:** Respondent ratings of e-intervention characteristic importance and selection/ranking of top five (*N* = 44)

Characteristic and brief definition	*N* (%) giving highest importance rating	*N* (%) placing in top 5	*N* (%) ranking as 1st or 2nd in importance
1. Behavioral target Drug use, depression, etc.	33 (75.0)	30 (68.2)	19 (43.2)
2. Change technique Personalized normed feedback, CBT, gamification	24 (54.5)	19 (43.2)	7 (15.9)
3. Type of technology Mobile, SMS, web	21 (47.7)	19 (43.2)	8 (18.2)
4. Level of counselor involvement None, some, extensive	21 (47.7)	12 (27.3)	6 (13.6)
5. Intended setting Health care, social services, schools, general	20 (45.5)	10 (22.7)	4 (9.1)
6. Population target Clinical, sub-threshold clinical, general	19 (43.2)	11 (25.0)	6 (13.6)
7. Theoretical basis None, some, extensive	19 (43.2)	11 (25.0)	6 (13.6)
8. Language Written in German, Mandarin, English?	18 (40.9)	4 (9.1)	2 (4.5)
9. Tailoring Extent of personalization based on user characteristics or preference	14 (31.8)	12 (27.3)	4 (9.1)
10. Number of sessions Single or multiple sessions, or ad libidem	13 (29.5)	10 (22.7)	0
11. Level of confidentiality Is user identifiable? Anonymous?	13 (29.5)	7 (15.9)	2 (4.5)
12. Cost structure Free, restricted, available?	12 (27.3)	10 (22.7)	2 (4.5)
13. Duration Amount of time sought per session/overall	12 (27.3)	7 (15.9)	2 (4.5)
14. Intention of impact Is goal to sustain, create, or prevent change?	12 (27.3)	6 (13.6)	1 (2.3)
15. Focus on therapeutic common factors Extent of attention to empathy, alliance?	12 (27.3)	0	0
16. Dynamic Does the intervention learn and adapt automatically?	13(25.0)	5 (11.4)	1 (2.3)
17. Centrality of visuals Visuals are primary, important, limited, absent?	11 (25.0)	2 (4.5)	0
18. Mode of data entry Data entry by self, clinician, sensor?	11 (25.0)	2 (4.5)	0
19. Level of counselor training needed, if applicable None, some, extensive?	11 (25.0)	0	0
20. Synchronous vs. asynchronous interactivity Fully synchronous, partially synchronous, or asynchronous responses?	9 (20.5)	2 (4.5)	0
21. Tunneling vs. surfing Free surfing vs. guiding user along a path	8 (18.2)	2 (4.5)	0
22. Text to convey information Text is central, important, limited, absent?	5 (11.4)	1 (2.3)	1 (2.3)
23. Focus Single or multi-focal?	4 (9.1)	1 (2.3)	0
24. Ethopoeia Lifelike characteristics, e.g., voice, narrator, personality	4 (9.1)	1 (2.3)	0
25. Audio to convey info Is audio primary, important, limited, absent?	4 (9.1)	0	0

Next, researchers who had been authors on articles related to e-health/m-health interventions in peer-reviewed journals indexed by PubMed, and for whom a valid email could be found, were emailed an invitation to complete the survey. A total of 123 people were contacted between November 11, 2015, and April 2, 2016. The survey was built and disseminated using the Qualtrics platform (www.Qualtrics.com). The Wayne State University Institutional Review Board (IRB) deemed this survey to be exempted from IRB review. The survey introduction noted that three randomly selected respondents would receive an Amazon gift card worth $100 or $50.

Participants were asked to evaluate these characteristics in three separate ways. First, they were asked to rate the importance of each characteristic as a key descriptor on a 1–5 scale, in which “1” indicated “very unimportant” and “5” indicated “very important.” Each descriptor included a brief definition (as in Table 1). Second, participants were asked to choose the five descriptors that they deemed most important in characterizing e-interventions, using the complete list of descriptors. Third, they were asked to rank those five descriptors in order of importance. Participants were also invited to contribute additional descriptors that were not included in the existing list.

From participant rankings, we selected items that received the highest importance rating from at least 25% of respondents, received a ‘top five’ ranking for at least 10% of participants, and received a first or second ranking from at least one participant. Two descriptors (“duration of time spent per session and/or overall” and “number of sessions”) were combined into one descriptor of “time spent”.

## Results

A total of 50 responses were received for a response rate of 40.7%. Six respondents who reported having little or no expertise in e-health research were removed from the dataset, leaving a final *N* of 44. These 44 respondents reported their discipline as psychology (22, 51.2%), public health (13, 30.2%), medicine (7, 16.3%), and social work (1, 2.3%). Of the 40 respondents who provided country of residence, 15 (37.5%) were from Sweden, 13 (32.5%) were from the USA, and between one and three respondents were from countries including Australia, Denmark, the Netherlands, Norway, Switzerland, and the UK.

Respondent ratings of each e-intervention characteristic are presented in Table 1. Given that the median was four for all but three characteristics, importance ratings for each intervention characteristic are presented in terms of the number and proportion of respondents giving each characteristic a rating of 5 for very important. Table 1 also provides the number and proportion of respondents who chose each characteristic as among the top five most important and the number and proportion of respondents ranking that item as either first or second in importance. Four e-intervention characteristics—behavioral target, change technique, type of technology, and level of counselor involvement—were ranked in the top four for all three ratings.

Eight participants suggested a total of 13 additional characteristics as important in describing e-interventions, eight of which were considered unique from the original list. One such characteristic, use of social media to communicate with others, was suggested by three participants. Additional characteristics were suggested once each and included automation, use of a fictive companion, type of hardware needed, level of interactivity, extent of scientific support, how clearly the intervention conveys a message regarding which behaviors to change, and how participants report on homework and training.

**Fig. 1:**
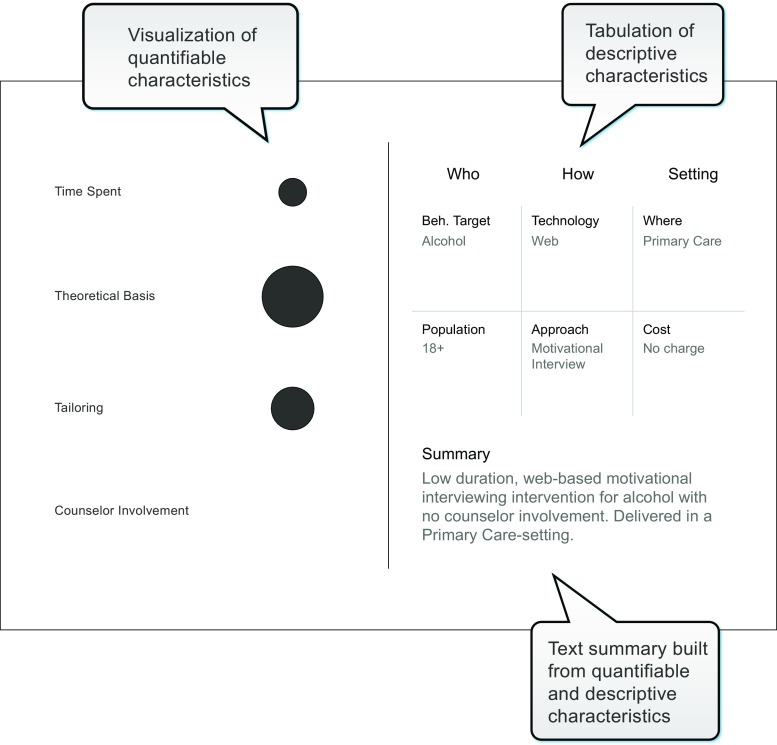
Depiction of visualization tool display of a single intervention

From Table 1, we identified ten characteristics that reached relative consensus about their importance when characterizing e-health/m-health interventions. Six characteristics were descriptive: behavioral target, target population, underpinning approach/change technique, type of technology, intended setting, and cost to user. Four characteristics were quantifiable: duration of the intervention, extent to which the intervention was informed by theory, extent of tailoring of content, and extent to which the intervention included counselor involvement. The visualization tool combines all ten characteristics and presents summary information in both graphical and text form (see Fig. 1).

## Discussion

The identification of salient characteristics from among a more comprehensive set of descriptors was borne out of the need for a tool to enable e-intervention developers and researchers to succinctly and consistently communicate the key characteristics of their intervention. The tool was developed to facilitate streamlined standardized characterization of e-health interventions, thereby establishing a basis for meaningful dialog across the diversity of e-health interventions.

Our results suggest that 10 specific intervention characteristics were consistently supported as of central importance by a panel of 44 e-intervention experts who reflected diversity of discipline and geographical location. These key characteristics are common features in review commentaries providing corroboration of meaningfulness and usefulness of the selected characteristics. The weight and perceived relevance of individual items differs across reviews; oftentimes, this difference is a result of the individual theoretical perspective and/or behavioral target of interest. The wording used by different studies may vary but essentially all are found to be central in previous work—“type of technology” [2, 5, 7, 10, 11], “change technique” [2, 3, 5, 7, 10], “behavior target” [5], and “level of counselor support” [3, 5, 11].

Our consensus with published literature points to the generalizability of the key characteristics identified here. Despite the similarities, our modeling of the key characteristics goes beyond previous efforts to develop guidelines, e.g., [5–7] by focusing on facilitating e-health intervention development. This facilitation has been achieved through identifying content of both a broader conceptual and more specific nature while not including contextual factors (such as health systems, security, regulations) and issues concerning outcome and reporting. By focusing on the design elements, they be of a broad or more specific nature, we seek to facilitate higher transparency and identification across e-health interventions, which may serve to accommodate future design of e-health interventions.

There is a clear need for standardized reporting of studies involving e-health interventions on elements such as methodological factors such as usability and content testing, extent of user feedback and cost assessment (e.g., [7]). There is also a clear need for comprehensive micro-level taxonomies (e.g., [2, 5, 10, 11]) that could inform meta-analyses and other broad analytic approaches. Neither of these important and ongoing efforts inform the need of intervention developers to report on a core set of key characteristics—long enough to be satisfactory at a high level but brief enough to be of practical use—in communications with other researchers. The visualization tool includes ten characteristics. Future research will investigate the possibility of reducing this number, the aim being to derive a tool that maintains practical use while being as succinct as possible.

Reviews provide an important synthesis of evidence addressing a specific domain; pan-review differences in describing interventions hinder the reader’s ability to gain a meaningful picture of how intervention characteristics differ within or across reviews. Future work will seek to further validate the checklist by (a) disseminating the checklist to elicit further input on its utility and content; (b) comparing multiple reviewers’ evaluations of existing interventions to establish inter-rater reliability and identify areas where greater definitional clarity is needed; and (c) collecting qualitative data from scenarios in which the checklist is used to facilitate rapid communication between intervention developers regarding key characteristics of their intervention. Notably, this approach to communication among e-intervention researchers may be uniquely amenable to crowd science approaches. We have initiated that direction by basing this initial draft on group input. We will continue in that vein by making available, at www.e-healthclassification.org, a series of visualization tools designed to facilitate characterization of an intervention in publications and presentations. This website is explicitly designed to facilitate group input regarding the characteristics and their visualization.

## Conclusion

The list of key characteristics included in the visualization tool was identified from a survey of e-health intervention experts. The first iteration of the visualization of salient characteristics represents an ambitious effort to develop a tool that will support communication of the defining characteristics for e-health interventions. The visualization tool will now be tested to enable an investigation into the effectiveness of the tool to enable communication.

The visualization tool aims to assist e-health developers and researchers to communicate the key characteristics of their interventions in a standardized manner that facilitates dialog. The tool is not designed to evaluate quality or effectiveness but rather to enable a standardized description of the intervention being developed and/or evaluated. This standardization will facilitate communication and comparison. This in turn will enable commonalities and differences to be uncovered, enabling learning from one intervention to the next to maximize the opportunity for interventions to be effective behavior change agents.
